# Hepatitis C virus Core overcomes all-*trans* retinoic acid-induced apoptosis in human hepatoma cells by inhibiting *p14* expression via DNA methylation

**DOI:** 10.18632/oncotarget.20337

**Published:** 2017-08-18

**Authors:** Juri Kwak, Jung-Hye Choi, Kyung Lib Jang

**Affiliations:** ^1^ Department of Microbiology, College of Natural Sciences, Pusan National University, Busan 609-735, Republic of Korea

**Keywords:** all-trans retinoic acid, apoptosis, hepatitis C virus Core, p53, p14

## Abstract

All-*trans* retinoic acid (ATRA), the most biologically active metabolite of vitamin A, is known to induce p14 expression via promoter hypomethylation to activate the p14-MDM2-p53 pathway, which leads to activation of the p53-dependent apoptotic pathway and subsequent induction of apoptosis in human hepatoma cells. In the present study, we found that hepatitis C virus (HCV) Core derived from ectopic expression or HCV infection overcomes ATRA-induced apoptosis in p53-positive hepatoma cells. For this effect, HCV Core upregulated both protein levels and enzyme activities of DNA methyltransferase 1 (DNMT1), DNMT3a, and DNMT3b and thereby repressed p14 expression via promoter hypermethylation, resulting in inactivation of the pathway leading to p53 accumulation in the presence of ATRA. As a result, HCV Core prevented ATRA from activating several apoptosis-related molecules, including Bax, p53 upregulated modulator of apoptosis, caspase-9, caspase-3, and poly (ADP-ribose) polymerase. In addition, complementation of p14 in the Core-expressing cells by either ectopic expression or treatment with 5-Aza-2′dC almost completely abolished the potential of HCV Core to suppress ATRA-induced apoptosis. Based on these observations, we conclude that HCV Core executes its oncogenic potential by suppressing the p53-dependent apoptosis induced by ATRA in human hepatoma cells.

## INTRODUCTION

Hepatitis C virus (HCV) is a major cause of acute and chronic hepatitis, which often leads to liver cirrhosis and hepatocellular carcinoma (HCC) [1]. As a member of the *Flaviviridae* family, HCV contains a positive stranded RNA genome of 9.5 kb, which encodes a polyprotein proteolytically processed into four structural proteins and six nonstructural proteins [2]. In addition to its role as a major component of nucleocapsid, HCV Core has been strongly implicated in HCC pathogenesis by virtue of its role in the alteration of diverse signaling pathways, transcriptional activation, modulation of immune responses, apoptosis, and lipid metabolism [3–5]. In addition, HCV Core has been directly implicated in cellular transformation and immortalization [6]. Furthermore, direct induction of HCC by HCV Core in transgenic mice has been reported [7]. Despite the steadily increasing knowledge about HCV Core in HCV-associated oncogenesis, its mechanism of action is still a controversial topic.

All-*trans* retinoic acid (ATRA), the most biologically active metabolite of vitamin A, is being increasingly included in both chemopreventive and therapeutic schemes for various tumoral diseases, including acute promyelocytic leukemia (APL) [8–10]. ATRA inhibits carcinogenesis by blocking the promotion of initiated or transformed cells by three mechanisms: induction of apoptosis, arrest of further growth of abnormal cells, and induction of the differentiation of abnormal cells back to normal cells. In particular, its potential to induce apoptosis has attracted interest in both clinical and basic studies [9, 11]. ATRA affects diverse pro- and anti-apoptotic molecules to induce apoptosis. For example, ATRA upregulates pro-apoptotic caspase-9 and Bax in breast cancer cells [12], but downregulates anti-apoptotic Bcl-2 and Survivin in neuroblastoma [13], melanoma [14], and myeloblastic leukemia cells [15]. ATRA also activates extrinsic apoptosis pathways through upregulation of tumor necrosis alpha (TNFα), caspase-8, and death receptor Fas [16–18]. Furthermore, it has been found that ATRA upregulates p53 and thereby activates several apoptosis-related molecules, including Bax, p53 upregulated modulator of apoptosis (PUMA), caspase-9, Bid, caspase-8, caspase-3, and poly (ADP-ribose) polymerase (PARP), which leads to apoptosis in human hepatoma cells [19]. These data indicate that ATRA-induced apoptosis involves activation of intrinsic and/or extrinsic apoptosis pathways.

HCV Core appears to exert its oncogenic activity, at least in part, by suppressing the anti-cancer activities of ATRA. For example, HCV Core overcomes ATRA-induced cell growth arrest by inhibiting *retinoic acid receptor-β2* (*RAR-β2*) expression via DNA methylation in human hepatoma cells [20]. However, it is unknown whether HCV Core suppresses ATRA-induced apoptosis. According to a recent report, ATRA activates p14 expression via promoter hypomethylation, which results in ubiquitin (Ub)-dependent proteasomal degradation of mouse double minute 2 (MDM2) and subsequent stabilization of p53, thus leading to the activation of the p53-dependent apoptotic pathway [19]. In addition, HCV Core is known to repress p14 expression via promoter hypermethylation [21]. These observations urged us to investigate whether HCV Core abolishes the potential of ATRA to induce apoptosis in human hepatoma cells by repressing p14 expression via promoter hypermethylation. Therefore, in the present study, we first examined whether HCV Core can overcome ATRA-induced apoptosis in p53-positive human hepatoma cells. Next, we examined whether HCV Core represses p14 expression via promoter hypermethylation and thereby inactivates the p14-MDM2-p53 pathway to downregulate p53 in the presence of ATRA. We then investigated whether HCV Core prevents ATRA from activating the p53-dependent apoptotic pathway and thus abolishes its potential to induce apoptosis in human hepatoma cells. Finally, we attempted to prove that HCV Core overcomes ATRA-induced apoptosis during HCV infection.

## RESULTS

### HCV Core overcomes ATRA-induced apoptosis in human hepatoma cells

**Figure 1:**
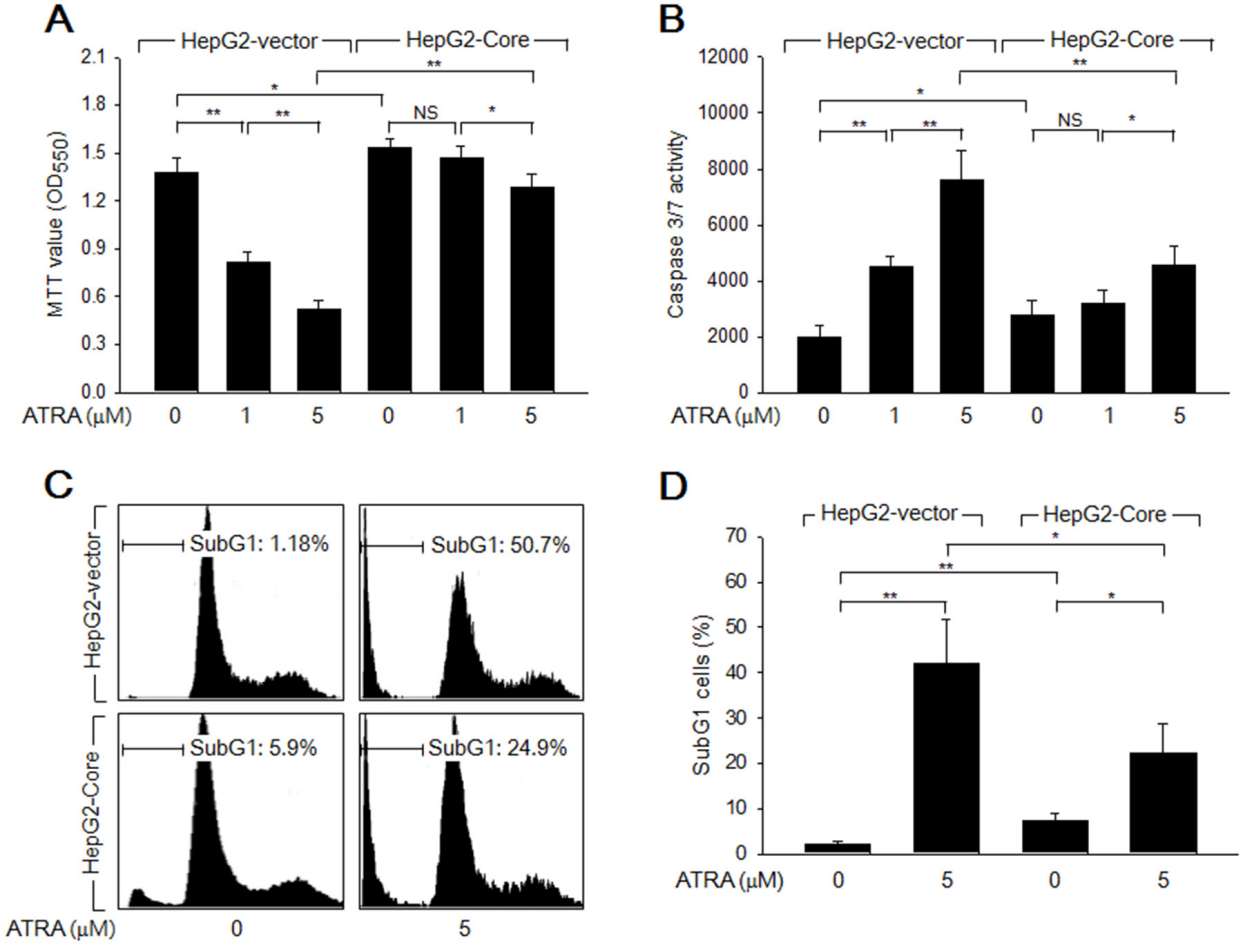
HCV Core overcomes ATRA-induced apoptosis in a human hepatoma cell line, HepG2 HepG2-vector and HepG2-Core cells were treated with ATRA at the indicated concentrations for 72 h. **(A)** MTT assay was performed to determine cell viability. Data represent the means ± SD of four independent experiments (n = 4). **(B)** The activities of caspase-3 and -7 were measured as described in the Methods section (n = 4). **(C)** Cell cycle profiles were determined by FACS analysis to show the subG1 fraction in percentage. **(D)** The graph shows the percentage of subG1 cells prepared as in (C) (n = 3).

Initially, we examined whether HCV Core stimulates the growth of HepG2 cells in the presence or absence of ATRA. Data from the 3-(4,5-dimethylthiazol-2-yl)-2,5-diphenyltetrazolium bromide (MTT) assay revealed that HCV Core significantly increased the number of viable cells (Figure 1A), which indicated that HCV Core stimulates hepatocyte growth, as previously demonstrated [22]. In addition, treatment with ATRA for 60 h dramatically reduced the viability of HepG2-vector cells in a dose-dependent manner, but the effect was relatively mild in HepG2-Core cells (Figure 1A); this result indicated that HCV Core abolishes the potential of ATRA to induce cell death in HepG2 cells. Based on these observations, we next investigated whether HCV Core overcomes ATRA-induced apoptosis in HepG2 cells. In the absence of ATRA, the activities of caspase-3 and -7 (potential apoptotic markers) in the HepG2-Core cells were slightly higher than those in the HepG2-vector cells (Figure 1B). Consistent with this result, HCV Core weakly induced apoptosis in HepG2 cells, as demonstrated by the increased subG1 fraction and terminal deoxynucleotidyl transferase (TdT)-mediated dUTP nick end labeling (TUNEL) positivity in the HepG2-Core cells (Figures 1C, 1D, 4E and 4F). Consistent with data from the MTT assay, we also found that ATRA dramatically increased the activities of caspase-3 and -7 in the HepG2-vector cells, whereas the effect was much weaker in the HepG2-Core cells (Figure 1B). Accordingly, ATRA substantially increased both the subG1 fraction and TUNEL positivity in the HepG2-vector cells, but the effects were negligible or mild in the HepG2-Core cells (Figures 1C, 1D, 4E and 4F). Taken together, these findings indicated that HCV Core abolishes the potential of ATRA to induce apoptosis in HepG2 cells, but that it also has an intrinsic proapoptotic activity in these cells.

**Figure 2:**
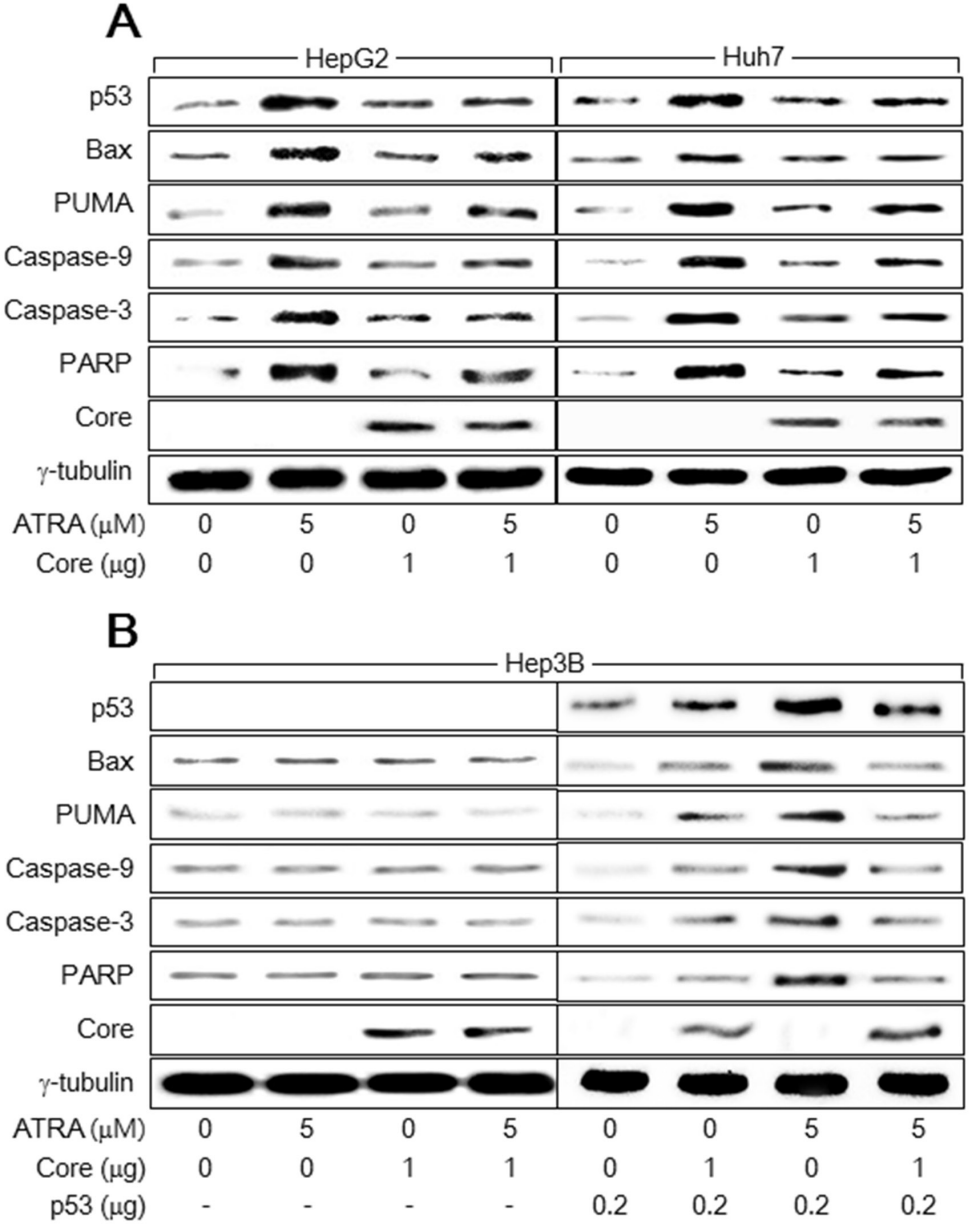
HCV Core suppresses the activation of the p53-dependent apoptotic pathway by ATRA HepG2, Huh7, and Hep3B cells were transiently transfected with either an empty vector or an HCV Core expression plasmid in the presence or absence of 5 μM ATRA for 48 h. **(A)** Levels of p53, Bax, PUMA, HCV Core, γ-tubulin, and active (cleaved) forms of caspase-9 (35 kDa), caspase-3 (20 kDa), and PARP (84 kDa) in the HepG2 and Huh7 cells were compared by western blotting. **(B)** Levels of the indicated proteins in the Hep3B cells were determined by western blotting. For lanes 5 to 8, the indicated amount of p53 expression plasmid was included in the transfection mixtures.

**Figure 3:**
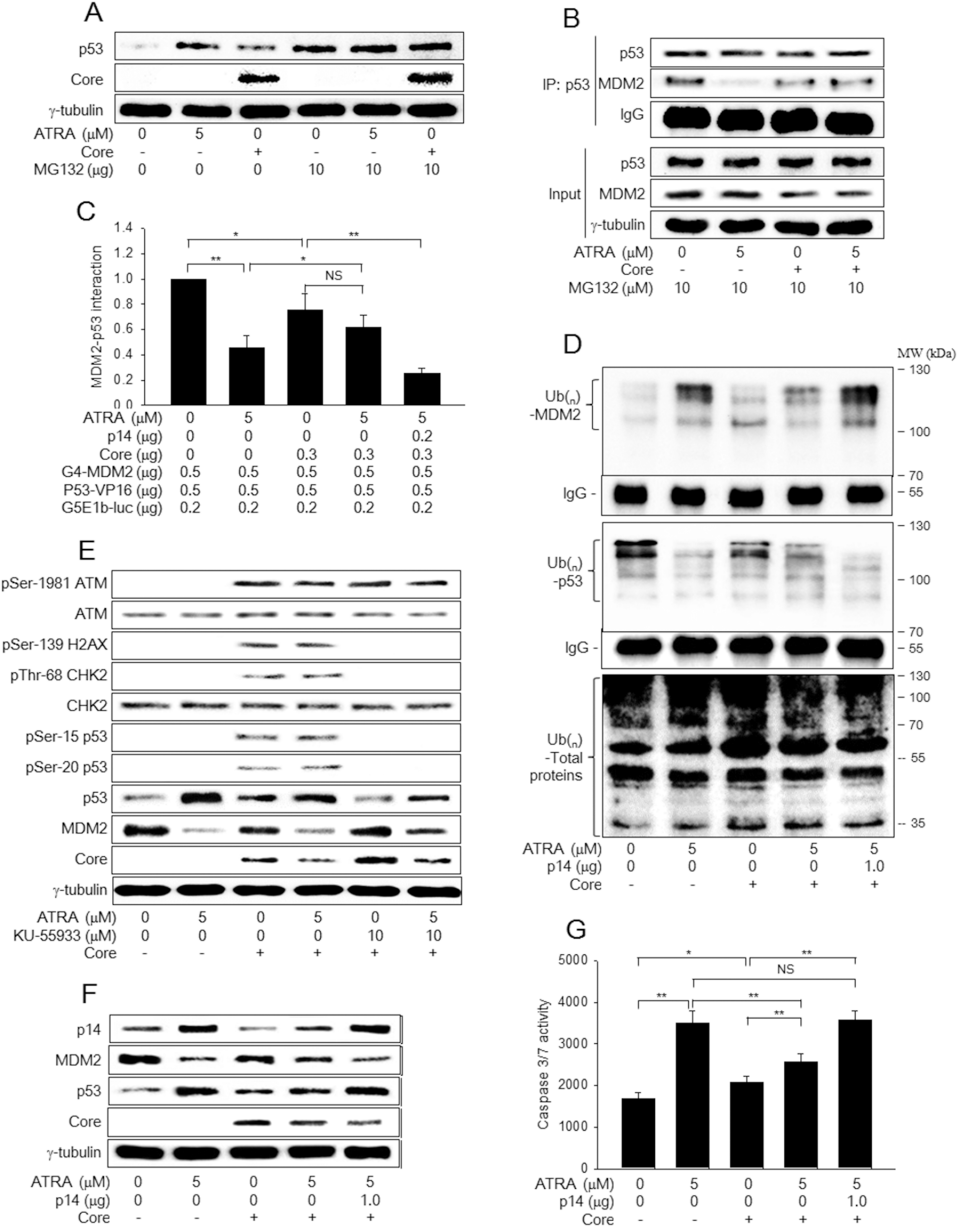
HCV Core inactivates the p14-MDM2 pathway to downregulate p53 levels in the presence of ATRA **(A)** HepG2-vector and HepG2-Core cells were either mock-treated or treated with 5 μM ATRA for 48 h. For lanes 4 to 6, cells were treated with 10 μM MG132 for 4 h before harvesting. Levels of p53, HCV Core, and γ-tubulin were measured by western blotting. **(B)** HepG2-vector and HepG2-Core cells were either mock-treated or treated with 5 μM ATRA for 44 h and then treated with 10 μM MG132 for additional 4 h. Total p53 protein was immunoprecipiated with an anti-p53 mouse monoclonal antibody and subjected to western blotting using an anti-p53 rabbit polyclonal antibody to detect p53 and an anti-MDM2 mouse monoclonal antibody to detect MDM2 and IgG. The input shows total p53 and MDM2 levels in the cell lysates. **(C)** The effects of ATRA and HCV Core on the interaction between MDM2 and p53 were determined by a mammalian two-hybrid assay. HepG2 cells were transiently transfected with the indicated plasmids and then treated with ATRA for 48 h, followed by the luciferase assay (n = 5). **(D)** HepG2-vector and HepG2-Core cells were transfected with pHA-Ub for 48 h in the presence or absence of 5 μM ATRA. For lane 5, the p14 expression plasmid was included. Total MDM2 and p53 proteins were immunoprecipitated with an appropriate antibody and subjected to western blotting using an anti-HA antibody to detect Ub-complexed MDM2 and p53 (upper and middle panels). Total ubiquitinated proteins in the cell extracts were detected by western blotting using an anti-Ub antibody (lower panel). **(E)** HepG2-vector and HepG2-Core cells were either mock-treated or treated with 5 μM ATRA for 48 h, followed by western blotting. For lanes 5 and 6, the cells were treated with 10 μM KU-55933 for 1 h before harvesting. **(F)** HepG2-vector and HepG2-Core cells were either mock-treated or treated with 5 μM ATRA for 48 h, followed by western blotting. For lane 5, the p14 expression plasmid was included. **(G)** The activities of caspase-3 and -7 were measured in cells prepared as in (F) (n = 4).

**Figure 4:**
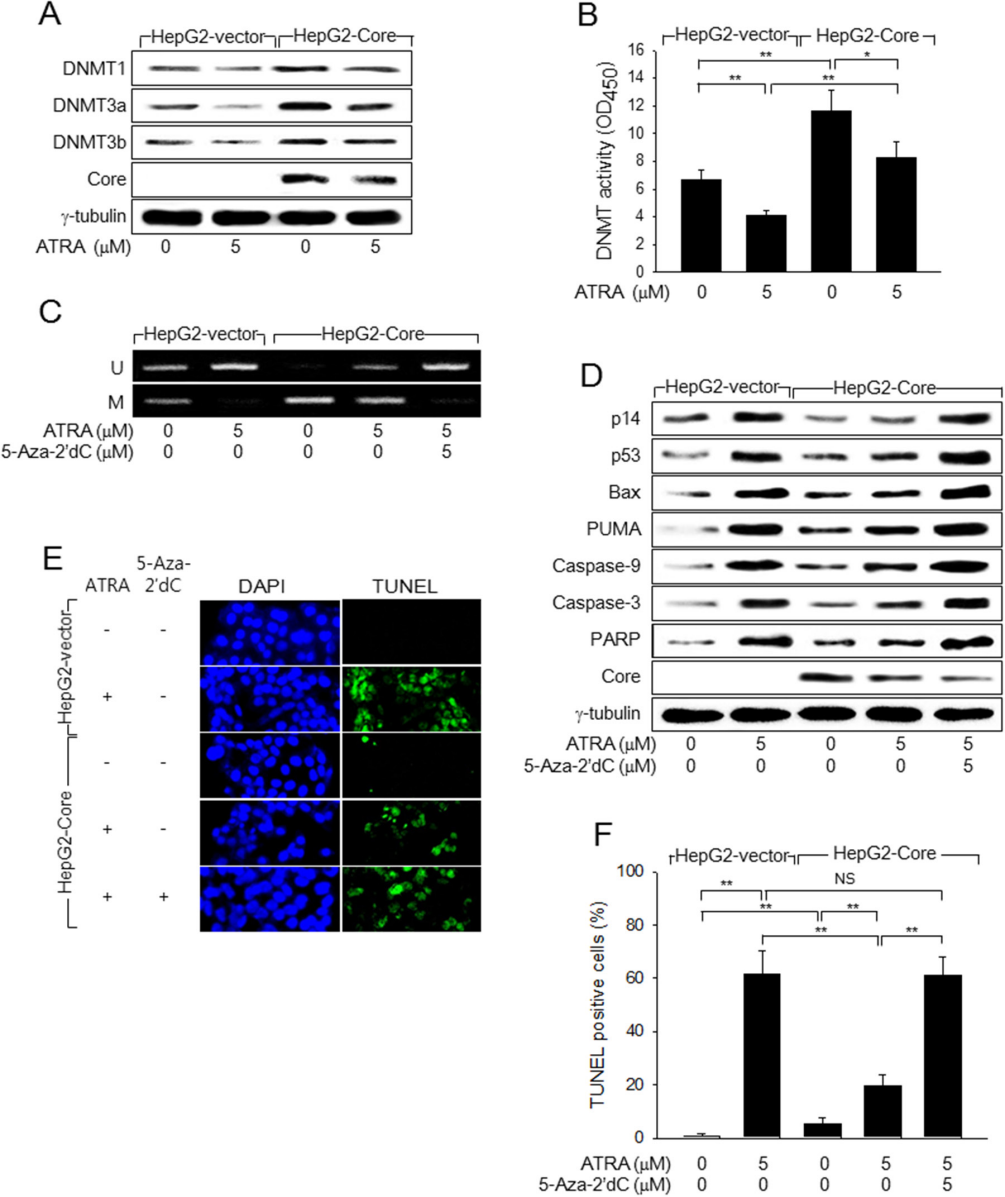
HCV Core represses p14 expression via promoter hypermethylation to overcome ATRA-induced apoptosis **(A)** HepG2-vector and HepG2-Core cells were either mock-treated or treated with 5 μM ATRA for 48 h, followed by western blotting. **(B)** DNMT activity in the cells prepared as in (A) was measured as described in the Methods section (n = 4). **(C)** Genomic DNA isolated from HepG2-vector and HepG2-Core cells prepared as in (A) was subjected to MSP analysis to determine whether the p14 promoter was methylated (M) or unmethylated (U). For lane 5, the cells were treated with 5-Aza-2′dC for 24 h before harvesting. **(D)** HepG2-vector and HepG2-Core cells prepared as in (C) were subjected to western blotting to measure levels of p14, p53, Bax, PUMA, HCV Core, γ-tubulin, and active (cleaved) forms of caspase-9, caspase-3, and PARP. **(E)** HepG2-vector and HepG2-Core cells were either mock-treated or treated with 5 μM ATRA for 72 h, followed by TUNEL assay. Total nuclei stained with DAPI (blue) and TUNEL-positive nuclei (green) are shown. **(F)** The graph shows the percentage of TUNEL-positive cells prepared as in (E) (n = 4).

### HCV Core prevents ATRA from activating the p53-dependent apoptotic pathway in human hepatoma cells

We next investigated the mechanism by which HCV Core overcomes ATRA-induced apoptosis in hepatocytes. ATRA was recently reported to activate the p53-dependent apoptotic pathway to induce apoptosis in human hepatic cells [19]. Consistent with this report, our findings showed that treatment with ATRA upregulated p53 and subsequently activated several apoptosis-related molecules including Bax, PUMA, caspase-9 and -3, and PARP in the HepG2 and Huh7 cells (Figure 2A, lanes 2 and 6). Moreover, HCV Core also slightly upregulated p53 to activate the p53-dependent apoptotic pathway in both HepG2 and Huh7 cells (Figure 2A, lanes 3 and 7), which was also consistent with previous reports [21, 23, 24]. However, neither ATRA nor HCV Core activated the apoptotic pathway in Hep3B cells, in which p53 was absent (Figure 2B, lanes 2 and 3). To confirm that p53 is critical for activation of the apoptotic pathway by ATRA and HCV Core, we attempted to complement p53 in Hep3B cells using a p53 expression plasmid. As a result, both ATRA and HCV Core upregulated ectopic p53 levels and thereby successfully activated the apoptotic pathway in Hep3B cells (Figure 2B, lanes 6 and 7). These results indicated that ATRA and HCV Core induce apoptosis via a common route, namely the p53-dependent apoptotic pathway. Interestingly, the potential of ATRA to upregulate p53 and subsequently activate the p53-dependent apoptotic pathway in HepG2 and Huh7 cells was severely impaired in the presence of HCV Core (Figure 2A, lanes 4 and 8). HCV Core also downregulated ectopic p53 levels in the presence of ATRA, resulting in impaired activation of the apoptotic pathway in Hep3B cells (Figure 2B, lanes 8). These results suggested that HCV Core prevents ATRA from upregulating p53 and thereby abolishes its potential to activate the 53-dependent apoptotic pathway in human hepatoma cells.

### HCV Core prevents ATRA from activating the p14-MDM2-p53 pathway

As shown in Figure 2, HCV Core abolishes the potential of ATRA to upregulate p53, although it has an intrinsic ability to upregulate p53. In general, p53 levels are negatively regulated by an E3 Ub ligase, MDM2, which induces ubiquitination and subsequent proteasomal degradation of p53 [25]. The MDM2 protein itself is also negatively regulated via Ub-dependent proteasomal degradation pathways [25, 26]. Therefore, we investigated whether ATRA and HCV Core upregulate p53 levels by affecting its proteasomal degradation. Treatment with a proteasomal inhibitor, MG132, almost completely abolished the potential of ATRA and HCV Core to upregulate p53 levels in HepG2 cells (Figure 3A, lanes 5 and 6), indicating that ATRA and HCV Core individually stabilize p53 by inhibiting its proteasomal degradation. In addition, data from the co-immunoprecipitation experiment (co-IP) and mammalian two-hybrid assay clearly revealed that ATRA and HCV Core decreased the interaction between p53 and MDM2 (Figure 3B, lanes 2 and 3; Figure 3C, columns 2 and 3). Moreover, both ATRA and HCV Core increased the levels of the ubiquitinated forms of MDM2 but decreased those of p53, without inducing dramatic changes in the ubiquitination patterns of other proteins (Figure 3D, lanes 2 and 3). The effects were more dramatic with ATRA, consistently with its potential to regulate p53 and MDM2 (Figure 3E and 3F). Interestingly, the potential of ATRA to interrupt the interaction between MDM2 and p53 was impaired in the presence of HCV Core (Figure 3B, lane 4; Figure 3C, column 4). Consistent with this, in the presence of ATRA, levels of the ubiquitinated forms of MDM2 were higher in the HepG2-vector cells whereas those of p53 were higher in the HepG2-Core cells (Figure 3D, lanes 2 and 4), resulting in MDM2 upregulation and p53 downregulation in HepG2-Core cells (Figure 3E and 3F, lane 4).

In general, both p53 ubiquitination and its protein stability are altered in response to cellular stresses such as DNA damage and oncogene activation, which lead to the activation of ataxia-telangiectasia mutated (ATM) and p14, respectively [27, 28]. Therefore, we first examined whether ATRA activates ATM to upregulate p53, whereas HCV Core differentially regulates ATM depending on the presence of ATRA. Consistent with previous reports [29, 30], HCV Core induced the phosphorylation of ATM at the Ser-1981 residue without affecting its total protein levels, which led to the phosphorylation of p53 at the Ser-15 residue (Figure 3E, lane 3). The activated ATM then induced the phosphorylation of CHK2 at the Thr-68 residue, a specific marker for ATM activation, without affecting its total protein levels, which led to the phosphorylation of p53 at the Ser-20 residue in the HepG2-Core cells. Phosphorylation of p53 at either the Ser-15 or the Ser-20 residue is known to increase its protein stability [28]; therefore, it is likely that HCV Core upregulates p53 by activating the ATM-CHK2 pathway in the absence of ATRA. Consistent with this, we also found that treatment with an ATM inhibitor, KU-55933, almost completely abolished the potential of HCV Core to upregulate p53 (Figure 3E, lane 5). Unlike HCV Core, ATRA had little effect on the ATM pathway in both HepG2-vector and HepG2-Core cells (Figure 3E, lanes 2 and 4), indicating that neither the ATRA-induced p53 activation nor its suppression by HCV Core involves the ATM-CHK2 pathway.

Next, we examined whether ATRA activates p14 expression to upregulate p53 while HCV Core represses it to reverse the effect of ATRA on p53. Consistent with a previous report [19], we found that ATRA upregulated p14 in the HepG2-vector cells, which led to the downregulation of MDM2 and subsequent upregulation of p53 in these cells (Figure 3F, lane 2). In contrast, HCV Core downregulated p14 both in the presence and absence of ATRA (Figure 3F, lanes 3 and 4). Despite p14 downregulation, HCV Core lowered MDM2 levels and elevated p53 levels in the absence of ATRA (Figure 3F, lane 3), presumably due to activation of the ATM-CHK2 pathway (Figure 3E, lane 3). In the presence of ATRA, however, HCV Core elevated MDM2 levels and lowered p53 levels (Figure 3F, lane 4), possibly via downregulation of p14. These results suggested that HCV Core exerts opposite effects on p53 in the presence and absence of ATRA by differentially affecting the ATM-CHK2 and p14-MDM2 pathways.

To prove that HCV Core actually downregulates p53 in the presence of ATRA via downregulation of p14, we attempted to ectopically complement p14 in the HepG2-Core cells. Ectopic expression of p14 increased the potential of ATRA to interrupt the interaction between MDM2 and p53 (Figure 3C, column 5). As a result, ectopic p14 expression increased the levels of the ubiquitinated forms of MDM2 but decreased those of p53 in the HepG2-Core cells in the presence of ATRA (Figure 3D, lane 5), which resulted in the downregulation of MDM2 and subsequent upregulation of p53 (Figure 3F, lane 5). Moreover, ectopic p14 expression almost completely abolished the potential of HCV Core to downregulate the activities of caspase-3 and -7 in the presence of ATRA (Figure 3G, column 5). Taken together, these findings indicated that HCV Core abolishes the potential of ATRA to activate the p53-dependent apoptotic pathway by downregulating p14.

### HCV Core prevents ATRA from inducing p14 expression via promoter hypomethylation

Next, we investigated how ATRA and HCV Core oppositely regulate p14 expression in human hepatoma cells. According to previous reports, ATRA induces p14 expression via promoter hypomethylation, whereas HCV Core represses it via promoter hypermethylation [19, 21]. Consistent with this, we found that ATRA downregulated DNMT1, DNMT3a, and DNMT3b and their enzyme activities to induce p14 promoter hypomethylation in the HepG2-vector cells (Figure 4A and 4C, lane 2; Figure 4B, column 2). In contrast, HCV Core increased the protein levels and enzyme activity of DNMTs to induce p14 promoter hypermethylation (Figure 4A and 4C, lane 3; Figure 4B, column 3). Therefore, it was reasonable to hypothesize that HCV Core represses p14 expression via promoter hypermethylation in the presence of ATRA. Indeed, we found that HCV Core could increase both the protein levels and the enzyme activities of DNMTs to induce p14 promoter hypermethylation in the presence of ATRA (Figure 4A and 4C, lane 4; Figure 4B, column 4). Moreover, treatment with a universal DNMT inhibitor, 5-Aza-2′dC, almost completely abolished the potential of HCV Core to repress p14 expression via promoter hypermethylation in the presence of ATRA (Figure 4C and 4D, lane 5), which enabled ATRA to activate the p53-dependent apoptotic pathway in the HepG2-Core cells (Figure 4D, lane 5). As a result, ATRA could effectively induce apoptosis in the HepG2-Core cells in the presence of 5-Aza-2′dC (Figure 4E and 4F). Taken together, these results indicated that HCV Core abolishes the potential of ATRA to induce apoptosis in HepG2 cells by repressing p14 expression via promoter hypermethylation.

**Figure 5:**
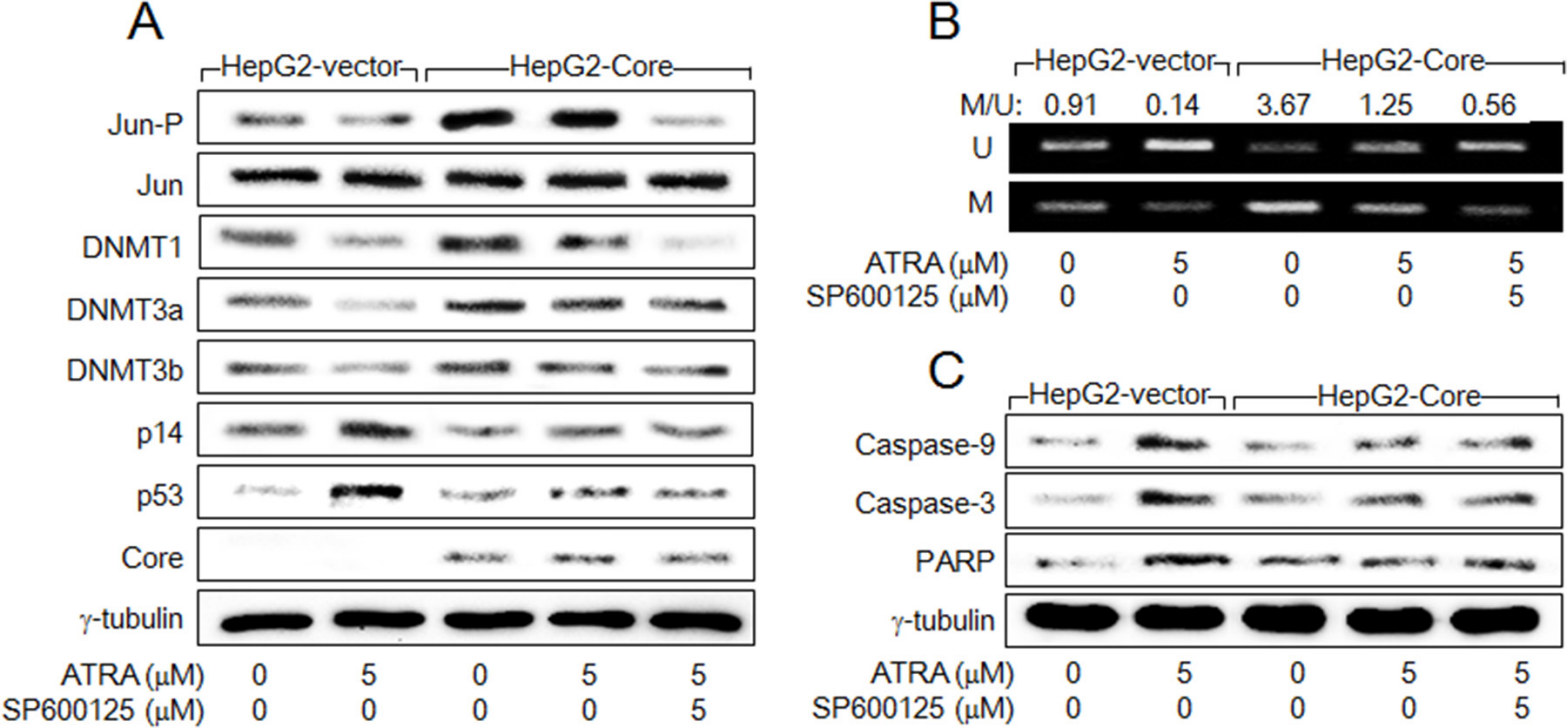
HCV Core activates DNMT1 expression in the presence of ATRA via activation of AP-1 HepG2-vector and HepG2-Core cells were either mock-treated or treated with ATRA for 48 h in the presence or absence of SP600125. **(A)** Levels of the indicated proteins were measured by western blotting. **(B)** The DNA methylation pattern of p14 promoter was analyzed by MSP as in Figure 4C. To present the ratio of methylated p14 to unmethylated p14 in the corresponding sample, DNA bands were quantified with the use of ImageJ image processing and analysis software (NIH). **(C)** Levels of the indicated proteins were measured by western blotting.

The mechanism by which ATRA and HCV Core oppositely regulate DNMT levels was investigated. It has been previously reported that the DNMT1 promoter contains the binding motifs of the AP-1 complex [31] and HCV Core activates DNMT1 expression via activation of AP-1 [32], while the promoters of DNMT3a and DNMT3b and their regulation by HCV Core are poorly understood. Consistently, HCV Core increased the phosphorylated form of c-Jun both in the presence and absence of ATRA, without affecting the total protein level of c-Jun (Figure 5A, lanes 3 and 4). In contrast, ATRA decreased the phosphorylated form of c-Jun in the absence of HCV Core (Figure 5A, lane 2). These results suggested that HCV Core and ATRA oppositely regulate DNMT1 expression via a common pathway involving c-Jun N-terminal kinases (JNKs). Indeed, treatment with a specific JNK inhibitor, SP600125, impaired the phosphorylation of c-Jun in the HCV Core-expressing cells and thereby abolished the potential of HCV Core to upregulate DNMT1 in the presence of ATRA, without affecting DNMT3a and DNMT 3b (Figure 5A, lane 5). However, treatment with SP600125 minimally affected the potential of HCV Core to induce p14 promoter hypermethylation (Figure 5B, lane 5), to downregulate p14 and p53 levels (Figure 5A, lane 5), and to suppress ATRA activation of the p53-dependent apoptotic pathway (Figure 5C, lane 5). These results suggested that DNMT3a and DNMT3b in addition to DNMT1 are involved in the repression of p14 expression and subsequent suppression of ATRA-induced apoptosis by HCV Core.

### HCV Core abolishes the potential of ATRA to induce apoptosis during virus replication

**Figure 6:**
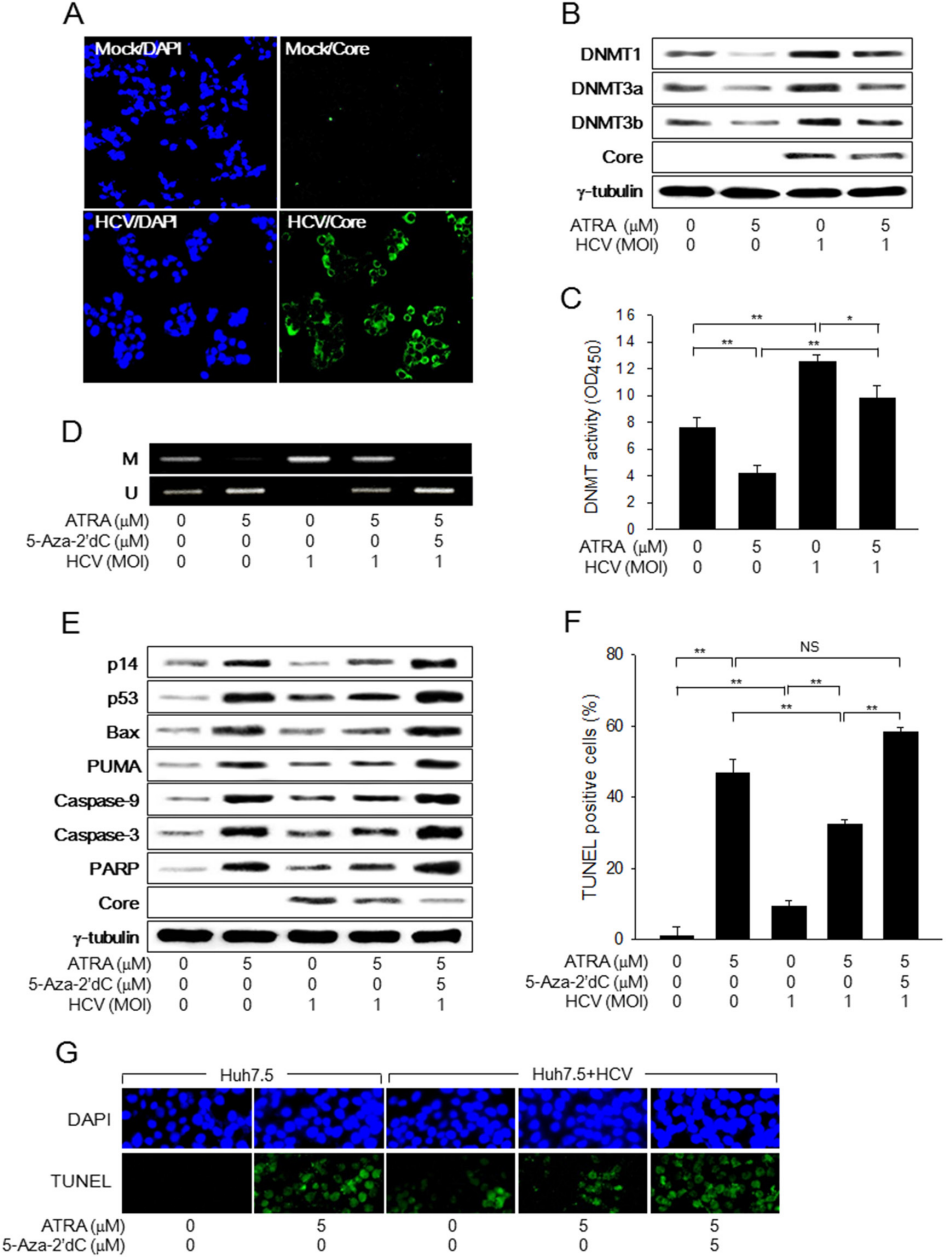
HCV Core overcomes ATRA-induced apoptosis during virus infection Huh7.5 cells were either mock-infected or infected with the JFH-1 strain of HCV at an MOI of 1.0 for 48 h in the presence or absence of 5 μM ATRA. **(A)** Nuclei (blue) and HCV Core (green) in the mock-infected and HCV-infected Huh7.5 cells were detected by DAPI staining and immunofluorescence assay, respectively. **(B)** Levels of the indicated proteins were measured by western blotting. **(C)** DNMT activity was determined as described in the Methods section (n = 4). **(D)** Genomic DNA isolated from cells was subjected to MSP analysis to determine the methylation status of the p14 promoter. For lane 5, the cells were treated with 5 μM 5-Aza-2′dC for 24 h before harvesting. **(E)** Cells were treated with 5-Aza-2′dC as described in Figure 4C and levels of the indicated proteins were measured by western blotting. **(F)** The graph shows the percentage of TUNEL-positive cells prepared as in (G) (n = 4). **(G)** Upper panels show total nuclei (blue) detected by DAPI staining and lower panels show TUNEL-positive nuclei (green) in Huh7.5 cells prepared as in (E).

Finally, we investigated whether HCV Core overcomes ATRA-induced apoptosis during HCV infection. When the JFH-1 strain of HCV was administered to Huh7.5 cells at an MOI of 1.0 for 48 h, it was possible to obtain a high infection rate (over 90%), as determined by immunofluorescence assay to detect HCV Core in the infected cells (Figure 6A). Under the conditions, HCV increased both the protein levels and the enzyme activities of DNMTs to induce p14 promoter hypermethylation, which led to the downregulation of p14 in the Huh7.5 cells (Figure 6B to 6E). Despite p14 downregulation, p53 levels were upregulated, possibly via activation of the ATM-CHK2 pathway, as demonstrated by ectopic HCV Core expression (Figure 3E), which resulted in the activation of the p53-dependent apoptotic pathway and subsequent induction of apoptosis in the HCV-infected cells (Figure 6E to 6G). Therefore, it was possible to reproduce the potential of HCV Core to induce apoptosis during HCV infection in Huh7.5 cells.

Treatment with ATRA decreased both the protein levels and the enzyme activities of DNMTs and thereby induced p14 promoter hypomethylation, which resulted in the upregulation of p14 in Huh7.5 cells (Figure 6B to 6E), as in HepG2 cells (Figure 4A to 4D). Accordingly, ATRA strongly upregulated p53 to activate the p53-dependent apoptotic pathway, thus resulting in dramatic induction of apoptosis in Huh7.5 cells (Figure 6E to 6G). The potentials of ATRA to induce p14 expression via promoter hypomethylation and induce apoptosis in Huh7.5 cells via activation of the p53-apoptotic pathway were severely impaired in the HCV-infected cells (Figure 6B to 6G). Moreover, treatment with 5-Aza-2’dC almost completely recovered the impaired potentials of ATRA in the HCV-infected cells (Figure 6D to 6G). Based on these observations, we concluded that HCV Core suppresses the apoptosis induced by ATRA during virus replication by antagonizing the potentials of ATRA to activate p14 expression via promoter hypomethylation and subsequently induce apoptosis via activation of the p53-apoptotic pathway.

## DISCUSSION

HCV infection is characterized by inflammatory liver damage called hepatitis and a long viral persistence associated with an increased risk of developing HCC [1, 2, 33]. Aberrant regulation of apoptosis by HCV infection has been implicated in both liver damage and HCC development, although the mechanisms underlying these apparently opposite processes are poorly understood. Liver damage associated with HCV infection is largely induced by host immune responses and in part by the direct cytopathic effects of HCV [34, 35]. In addition, there is increasing evidence indicating that the processes of neoplastic transformation, progression, and metastasis involve alterations in the normal apoptotic pathways [36, 37]. There is increasing evidence that the processes of neoplastic transformation, progression and metastasis involve alterations in the normal apoptotic pathways. There is increasing evidence that the processes of neoplastic transformation, progression and metastasis involve alterations in the normal apoptotic pathways The roles of individual HCV proteins either in cultured cells or in transgenic animal models are also contradictory, exhibiting both pro- and anti-apoptotic effects. For instance, HCV Core inhibits CD95-, cisplatin-, c-Myc- and TNFα-induced caspase activation and apoptosis in cultured cells [38–40]. However, it has also been reported that cells expressing HCV Core are more prone to undergo apoptosis in response to anti-CD95 and serum starvation [41–43]. Consistent with these reports, in the present study, we found that HCV Core induces apoptosis by itself, but suppresses the apoptosis induced by ATRA in human hepatoma cells. Therefore, we attempted to uncover the mechanisms underlying the dual roles of HCV Core in the regulation of apoptosis in the presence and absence of ATRA.

Cells committed to suicide via p53-dependent apoptosis typically follow the mitochondrial pathway [44], but p53 can also induce cell death through death receptors [45]. The key role of p53 in apoptosis is primarily dependent on its transcriptional activity. For example, p53 can activate the transcription of various pro-apoptotic genes including those encoding members of the Bcl-2 family, such as the BH-3 only proteins Bax, Noxa, and PUMA, which target the mitochondria to induce cytochrome c release and subsequent activation of caspase-9, leading to activation of the intrinsic apoptotic pathway [44]. In the present study, we found that HCV Core slightly upregulates p53 levels to induce marginally intrinsic apoptosis in human hepatoma cell lines such as HepG2 and Huh7.5. In contrast, ATRA could strongly upregulate p53 levels, which induced more drastic apoptosis in these cells. Interestingly, the potentials of ATRA to upregulate p53 levels and thereby activate the p53-dependent apoptotic pathway were severely impaired in the presence of HCV Core. Accordingly, HCV Core-expressing cells were significantly resistant to apoptosis induced by ATRA. It is thus likely that HCV Core exhibits both anti- and pro-apoptotic activities in human hepatic cells by oppositely affecting the p53-dependent apoptotic pathway depending on the presence or absence of ATRA.

Previous reports have shown that HCV Core targets the p53 pathway via at least three different means: physical interaction, post-translational modifications, and modulation of p53 regulatory pathways [46]. First, HCV Core directly binds to p53 to either inhibit or activate it, thus resulting in its anti- or pro-apoptotic effects [23, 24, 46]. In addition, HCV Core enhances the transcriptional activity of p53 by affecting its posttranslational modifications [46]. HCV Core also counteracts p53-mediated growth suppression through activation of the MAPK and PI3K/Akt pathways, which enhances cell growth and proliferation [47]. In the present study, we found that HCV Core upregulates p53 via activation of the ATM-CHK2 pathway but downregulates p53 in the presence of ATRA via inactivation of the p14-MDM2 pathway, which is consistent with its dual roles in the regulation of the p53-dependent apoptotic pathway.

**Figure 7:**
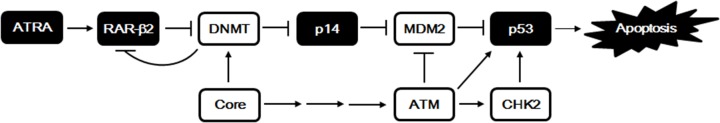
A proposed model for the induction of p53-dependent apoptosis by ATRA and its suppression by HCV Core ATRA in combination with RAR-β2 downregulates both the protein levels and the enzyme activities of DNMT1, DNMT3a, and DNMT3b via unknown mechanisms, resulting in activation of p14 expression via promoter hypomethylation. RAR-β2 complexed with ATRA also induces hypomethylation of its own promoter, thus amplifying its expression via a positive feedback loop. The elevated p14 levels then induce degradation of MDM2 via the Ub-proteasome system, thus leading to the stabilization of p53 and subsequent induction of apoptosis. In contrast, HCV Core antagonizes ATRA by increasing both the protein levels and the enzyme activities of DNMTs and represses expression of p14 and *RAR-β2* genes via promoter hypermethylation, resulting in suppression of ATRA-induced apoptosis. HCV Core also can upregulate p53 levels by activating ATM and CHK2 to marginally induce apoptosis in the absence of ATRA.

Based on the present study and other previous studies, we propose a mechanism by which HCV Core differentially regulates apoptosis depending on the presence or absence of ATRA (Figure 7). Several reports have proposed that HCV infection causes DNA breaks, which increases the mutation frequency of cellular genes, and HCV Core itself is a potent reactive oxygen species inducer that can cause DNA damage [30, 48], which suggests that HCV Core activates p53 via modulation of the DNA damage signaling pathway. Consistent with this, our results showed that HCV Core successively activates ATM and CHK2, which in turn induces p53 phosphorylation, thus resulting in its accumulation. In addition, ATM phosphorylation of MDM2 is also involved in robust p53 stabilization upon DNA damage [49]. For the negative regulation of p53, HCV Core downregulates p14 via DNA methylation to inactivate the p14-MDM2 pathway. For this effect, HCV Core increases both the protein levels and the enzyme activities of DNMT1, DNMT3a, and DNMT3b, thus resulting in promoter hypermethylation of the p14 gene. However, HCV Core cannot downregulate p53 levels in the absence of ATRA, possibly because the positive regulation of p53 by HCV Core via activation of the ATM-CHK2 pathway overrides the negative regulation of the p14-MDM2 pathway via p14 downregulation. Unlike HCV Core, ATRA activates p14 expression via promoter hypomethylation, which leads to destabilization of MDM2 and subsequent stabilization of p53 [19]. For this effect, ATRA in combination with RAR-β2 downregulates both the protein levels and the enzyme activities of DNMT1, DNMT3a, and DNMT3b [20], via unknown mechanisms. The present study suggests that ATRA represses DNMT1 expression via inactivation of AP-1. Therefore, it is likely that HCV Core and ATRA antagonize each other for the regulation of p53 via the p14-MDM2 pathway. Under our experimental conditions, HCV Core appears to be dominant over ATRA in the regulation of p14 expression via DNA methylation. However, it might be possible to postulate an opposite condition that makes ATRA override the potential of HCV Core to downregulate p53 levels. Indeed, treatment with ATRA at higher concentrations (over 10 μM) completely abolished the potential of HCV Core to overcome ATRA-induced apoptosis (data not shown).

The natural anti-cancer compound, ATRA, has been extensively investigated for the prevention and treatment of cancer, predominantly because of its ability to induce apoptosis in cells derived from various human cancers including APL, hepatoma, breast cancer, lung cancer, and head and neck cancer [9, 11, 12, 50, 51]. The present study also showed that ATRA induced apoptosis in human hepatoma cell lines, including HepG2 and Huh7, by activating the p53-dependent apoptotic pathway. In addition, ATRA could induce apoptosis in HCV Core-expressing cells, although the effect was not dramatic under our experimental conditions. On the other hand, considering the roles of apoptosis in preventing the production of new virus particles and in eliminating cells carrying harmful phenotypes that might lead to the development of cancer, the potential of HCV Core to overcome ATRA-induced apoptosis must be critical for its roles as a regulator of virus replication as well as a viral oncoprotein. Therefore, the antagonism between HCV Core and ATRA might be valuable in understanding the anti-cancer activities of ATRA as well as the oncogenic potential of HCV Core in HCV-infected human hepatoma cells.

## MATERIALS AND METHODS

### Plasmids

Plasmid pCMV-3 × HA1-Core encodes the full-length HCV Core downstream of three copies of the influenza virus HA epitope [52]. Plasmids pEGFP-N1-p14 (p14) and pHA-Ub were kindly provided by B.-J. Park (Pusan National University, Korea) and Y. Xiong (University of North Carolina at Chapel Hill, USA), respectively. Plasmids pG4-MDM2, pCMV-p53/VP16, and G5E1b-luc used for the mammalian two-hybrid assay [53] and pCMV-p53-WT were kindly provided by C.-W. Lee (Sungkyunkwan University, Korea).

### Cell culture and ATRA treatment

HepG2 (KCLB No. 88065), Hep3B (KCLB No. 88064), and Huh-7 (KCLB No. 60104) cells were obtained from the Korean Cell Line Bank. For transient expression, 2 × 10^5^ cells per 60-mm dish were transfected with 1 μg of appropriate plasmid(s) with the use of WelFect-EX PLUS (WelGENE) following the manufacturer’s instructions. Stable cell lines, HepG2-vector and HepG2-Core, were established by transfection with pCMV-3 × HA1 and pCMV-3 × HA1-Core, respectively, followed by selection with 500 μg/ml G418 (Gibco) [32]. For ATRA treatment, cells were incubated in medium containing ATRA (Sigma) or with only DMSO (Sigma). The concentration of ATRA in HepG2 cells has been reported to decline markedly over time as a direct consequence of both its degradation and metabolism [54]. To overcome this problem, the cells were refed with fresh medium containing DMSO or ATRA at intervals of 24 h.

### HCV infection system

The plasmid pJFH-1 containing HCV cDNA from a Japanese patient with fulminant hepatitis behind a T7 promoter [55] was linearized at the 3′ end of the HCV cDNA by *Xba*I digestion. The linearized DNA was then used as a template for *in vitro* transcription (MEGAscript; Ambion). Ten micrograms of JFH-1 RNA was delivered to Huh-7.5 cells by electroporation, and virus stocks were prepared as described by Zhong *et al.* [56]. For the determination of virus titer, real-time RT-PCR analysis was performed as described before [57]. Cells were either mock-infected or infected with HCV at a multiplicity of infection (MOI) of 1.0 under the indicated conditions.

### Cell viability analysis

For the determination of cell viability, the MTT assay was performed as described before [58]. Briefly, 5 × 10^3^ cells per well in 96-well plates were treated with an increasing concentration of ATRA for 60 h. The cells were then treated with 10 μM MTT (Sigma) for 4 h at 37°C. The formazan compounds derived from MTT by mitochondrial dehydrogenases of the living cells were then dissolved in DMSO, and quantified by measuring absorbance at 550 nm.

### Cell cycle analysis

Cell cycle profile was analyzed by flow cytometry. Briefly, 5 × 10^5^ cells were seeded in a 60-mm cell culture dish. The cells were then either mock-treated or treated with 5 μM ATRA for 60 h. Next, the cells were trypsinized, fixed in 80% ethanol, and resuspended in 50 μg/ml propidium iodide (Sigma) containing 125 U/ml RNase A (Sigma). DNA contents were analyzed by flow cytometry using the Cell-FIT software (Becton-Dickinson Instruments).

### TUNEL assay

Apoptotic cells were detected by TUNEL assay using the DeadEnd Fluorometric TUNEL System (Promega) according to the manufacturer’s instructions. Cells grown on coverslips were first fixed in 4% formaldehyde (Sigma) for 25 min at 4°C and then permeabilized in 0.2% Triton X-100 for 5 min. Next, the cells were incubated with TdT reaction mix for 1 h at 37°C and then with 2× SSC for 15 min to stop the reaction. Slides were prepared by adding UltraCruz mounting medium (Santa Cruz Biotechnology) containing 4', 6-diamidino-2-phenylindole (DAPI) and then visualized with an Eclipse fluorescence microscope (Nikon).

### Mammalian two-hybrid assay

Mammalian two-hybrid assay was performed to measure p53-MDM2 interactions, as previously described [19]. Briefly, 2 × 10^5^ cells per 60-mm diameter plate were transiently transfected with 0.2 μg of G5E1b-luc, 0.3 μg of an effector plasmid, 0.5 μg each of G4-MDM2 and p53-VP16, and 0.2 μg of pCH110 (Pharmacia) containing the *Escherichia coli* (*E. coli*) lacZ gene under control of the SV40 promoter for 48 h in the presence or absence of ATRA, followed by luciferase assay using a luciferase assay system (Promega). The luciferase value obtained was normalized to the β-galactosidase activity measured in the corresponding cell extract.

### Western blot analysis

Cells were lysed in buffer [50 mM Tris-HCl, pH 7.5, 150 mM NaCl, 0.1% SDS, 1% NP-40] supplemented with protease inhibitors. The cell extracts were then separated by SDS–PAGE and transferred onto a nitrocellulose membrane (Hybond PVDF, Amersham). The membranes were then incubated with antibodies against p14, pSer-1981 ATM, pSer-15 p53, pSer-20 p53 and pThr-68 CHK2 (Cell signaling), p53, Bax, caspase-3, caspase-9, c-Jun, phosphor-c-Jun, CHK2, HA, MDM2, PARP, PUMA, and Ub (Santa Cruz Biotechnology), HCV Core (Virogen), and γ-tubulin (Sigma). Primary antibodies were detected with the appropriate horseradish peroxidase (HRP)-conjugated secondary antibodies: anti-mouse IgG (H + L)-HRP (Bio-Rad), anti-goat IgG (H + L)-HRP (Bio-Rad), and anti-rabbit IgG (H + L)-HRP (Bio-Rad). The ECL kit (Amersham) was used to visualize protein bands via the ChemiDoc XRS imaging system (Bio-Rad).

### Immunoprecipitation (IP) assay

First, 2 × 10^6^ cells per 100-mm diameter plate were either mock-treated or treated with 5 μM ATRA for 60 h. The IP assay was then performed using the Classic Magnetic IP/Co-IP assay kit (Pierce) according to the manufacturer’s specifications. Briefly, the whole cell lysates (500 μg) were incubated overnight at 4°C with an appropriate IP antibody (2 μg). Pierce protein A/G magnetic beads (0.25 mg) were then added and incubated for additional 1 h. The beads were then collected using a magnetic stand (Pierce), and the antigen/antibody complexes eluted from them were subjected to western blotting.

### Caspase-3/7 activity assay

The activities of caspase-3 and -7 were measured using the Caspase-Glo 3/7 Assay (Promega). Briefly, 3 × 10^3^ cells seeded in 96-well plates were treated with ATRA as described above. Next, after adding an equal volume of Caspase-Glo-3 reagent (Promega) to each well, the mixtures were incubated for additional 1 h at room temperature, followed by measurement of luminescence.

### DMMT activity assay

First, 2 × 10^5^ cells per 60-mm diameter plate were either mock-treated or treated with 5 μM ATRA for 60 h. DNMT activity in the cell lysates was then measured using the EpiQuick DNMT Activity/Inhibition Assay Ultra Kit (Epigentek) following the manufacturer’s instructions.

### Methylation-specific PCR (MSP)

Genomic DNA (1 μg) denatured in 50 μl of 0.2 N NaOH was modified by treatment with 30 μl of 10 mM hydroquinone (Sigma) and 520 μl of 3 M sodium bisulfite (pH 5.0; Sigma) at 50°C for 16 h. For MSP, the modified DNA (100 ng) was amplified with *Taq* polymerase using both methylated and unmethylated primer pairs of p14 under the conditions described previously [59].

### Statistical analysis

Each experiment was repeated at least three times. The values represent means ± standard deviations (SDs). The difference between the means of the treatment group and the controls was assessed using the paired two-tailed *t*-test; the difference was considered significant if *P* < 0.05.

## References

[1] Tornesello ML, Buonaguro L, Izzo F, Buonaguro FM (2016). Molecular alterations in hepatocellular carcinoma associated with hepatitis B and hepatitis C infections. Oncotarget.

[2] Suzuki T, Aizaki H, Murakami K, Shoji I, Wakita T (2007). Molecular biology of hepatitis C virus. J Gastroenterol.

[3] Liang TJ, Heller T (2004). Pathogenesis of hepatitis C-associated hepatocellular carcinoma. Gastroenterology.

[4] Koike K (2007). Hepatitis C virus contributes to hepatocarcinogenesis by modulating metabolic and intracellular signaling pathways. J Gastroenterol Hepatol.

[5] Banerjee A, Ray RB, Ray R (2010). Oncogenic potential of hepatitis C virus proteins. Viruses.

[6] Ray RB, Lagging LM, Meyer K, Ray R (1996). Hepatitis C virus core protein cooperates with ras and transforms primary rat embryo fibroblasts to tumorigenic phenotype. J Virol.

[7] Moriya K, Fujie H, Shintani Y, Yotsuyanagi H, Tsutsumi T, Ishibashi K, Matsuura Y, Kimura S, Miyamura T, Koike K (1998). The core protein of hepatitis C virus induces hepatocellular carcinoma in transgenic mice. Nat Med.

[8] Hansen LA, Sigman CC, Andreola F, Ross SA, Kelloff GJ, De Luca LM (2000). Retinoids in chemoprevention and differentiation therapy. Carcinogenesis.

[9] Siddikuzzaman, Guruvayoorappan C, Berlin Grace VM (2011). All trans retinoic acid and cancer. Immunopharmacol Immunotoxicol.

[10] Okuno M, Kojima S, Matsushima-Nishiwaki R, Tsurumi H, Muto Y, Friedman SL, Noriwaki H (2004). Retinoids in cancer chemoprevention. Curr Cancer Drug Targets.

[11] Noy N (2010). Between death and survival: retinoic acid in regulation of apoptosis. Annu Rev Nutr.

[12] Donato LJ, Noy N (2005). Suppression of mammary carcinoma growth by retinoic acid: proapoptotic genes are targets for retinoic acid receptor and cellular retinoic acid-binding protein II signaling. Cancer Res.

[13] Niizuma H, Nakamura Y, Ozaki T, Nakanishi H, Ohira M, Isogai E, Kageyama H, Imaizumi M, Nakagawara A (2006). Bcl-2 is a key regulator for the retinoic acid-induced apoptotic cell death in neuroblastoma. Oncogene.

[14] Zhang H, Rosdahl I (2004). Expression profiles of p53, p21, bax and bcl-2 proteins in all-trans-retinoic acid treated primary and metastatic melanoma cells. Int J Oncol.

[15] Zheng A, Mantymaa P, Saily M, Savolainen E, Vahakangas K, Koistinen P (2000). p53 pathway in apoptosis induced by all-trans-retinoic acid in acute myeloblastic leukaemia cells. Acta Haematol.

[16] Jiang M, Zhu K, Grenet J, Lahti JM (2008). Retinoic acid induces caspase-8 transcription via phospho-CREB and increases apoptotic responses to death stimuli in neuroblastoma cells. Biochim Biophys Acta.

[17] Engedal N, Auberger P, Blomhoff HK (2009). Retinoic acid regulates Fas-induced apoptosis in Jurkat T cells: reversal of mitogen-mediated repression of Fas DISC assembly. J Leukoc Biol.

[18] Dhandapani L, Yue P, Ramalingam SS, Khuri FR, Sun SY (2011). Retinoic acid enhances TRAIL-induced apoptosis in cancer cells by upregulating TRAIL receptor 1 expression. Cancer Res.

[19] Heo SH, Kwak J, Jang KL (2015). All-trans retinoic acid induces p53-depenent apoptosis in human hepatocytes by activating p14 expression via promoter hypomethylation. Cancer Lett.

[20] Lee H, Woo YJ, Kim SS, Kim SH, Park BJ, Choi D, Jang KL (2013). Hepatitis C virus Core protein overcomes all-trans retinoic acid-induced cell growth arrest by inhibiting retinoic acid receptor-beta2 expression via DNA methylation. Cancer Lett.

[21] Seo YL, Heo S, Jang KL (2015). Hepatitis C virus core protein overcomes H2O2-induced apoptosis by downregulating p14 expression via DNA methylation. J Gen Virol.

[22] Fukutomi T, Zhou Y, Kawai S, Eguchi H, Wands JR, Li J (2005). Hepatitis C virus core protein stimulates hepatocyte growth: correlation with upregulation of wnt-1 expression. Hepatology.

[23] Lu W, Lo SY, Chen M, Wu K, Fung YK, Ou JH (1999). Activation of p53 tumor suppressor by hepatitis C virus core protein. Virology.

[24] Otsuka M, Kato N, Lan K, Yoshida H, Kato J, Goto T, Shiratori Y, Omata M (2000). Hepatitis C virus core protein enhances p53 function through augmentation of DNA binding affinity and transcriptional ability. J Biol Chem.

[25] Fang S, Jensen JP, Ludwig RL, Vousden KH, Weissman AM (2000). Mdm2 is a RING finger-dependent ubiquitin protein ligase for itself and p53. J Biol Chem.

[26] Inuzuka H, Fukushima H, Shaik S, Wei W (2010). Novel insights into the molecular mechanisms governing Mdm2 ubiquitination and destruction. Oncotarget.

[27] Bieging KT, Mello SS, Attardi LD (2014). Unravelling mechanisms of p53-mediated tumour suppression. Nat Rev Cancer.

[28] Lakin ND, Jackson SP (1999). Regulation of p53 in response to DNA damage. Oncogene.

[29] Banerjee A, Saito K, Meyer K, Banerjee S, Ait-Goughoulte M, Ray RB, Ray R (2009). Hepatitis C virus core protein and cellular protein HAX-1 promote 5-fluorouracil-mediated hepatocyte growth inhibition. J Virol.

[30] Machida K, Cheng KT, Sung VM, Lee KJ, Levine AM, Lai MM (2004). Hepatitis C virus infection activates the immunologic (type II) isoform of nitric oxide synthase and thereby enhances DNA damage and mutations of cellular genes. J Virol.

[31] Bigey P, Ramchandani S, Theberge J, Araujo FD, Szyf M (2000). Transcriptional regulation of the human DNA methyltransferase (dnmt1) gene. Gene.

[32] Park SH, Lim JS, Lim SY, Tiwari I, Jang KL (2011). Hepatitis C virus Core protein stimulates cell growth by down-regulating p16 expression via DNA methylation. Cancer Lett.

[33] Caselmann WH, Alt M (1996). Hepatitis C virus infection as a major risk factor for hepatocellular carcinoma. J Hepatol.

[34] Bantel H, Ruck P, Gregor M, Schulze-Osthoff K (2001). Detection of elevated caspase activation and early apoptosis in liver diseases. Eur J Cell Biol.

[35] Bantel H, Schulze-Osthoff K (2003). Apoptosis in hepatitis C virus infection. Cell Death Differ.

[36] Park YN, Chae KJ, Kim YB, Park C, Theise N (2001). Apoptosis and proliferation in hepatocarcinogenesis related to cirrhosis. Cancer.

[37] Fabregat I (2009). Dysregulation of apoptosis in hepatocellular carcinoma cells. World J Gastroenterol.

[38] Ray RB, Meyer K, Ray R (1996). Suppression of apoptotic cell death by hepatitis C virus core protein. Virology.

[39] Ray RB, Meyer K, Steele R, Shrivastava A, Aggarwal BB, Ray R (1998). Inhibition of tumor necrosis factor (TNF-alpha)-mediated apoptosis by hepatitis C virus core protein. J Biol Chem.

[40] Marusawa H, Hijikata M, Chiba T, Shimotohno K (1999). Hepatitis C virus core protein inhibits Fas- and tumor necrosis factor alpha-mediated apoptosis via NF-kappaB activation. J Virol.

[41] Honda M, Kaneko S, Shimazaki T, Matsushita E, Kobayashi K, Ping LH, Zhang HC, Lemon SM (2000). Hepatitis C virus core protein induces apoptosis and impairs cell-cycle regulation in stably transformed Chinese hamster ovary cells. Hepatology.

[42] Ruggieri A, Harada T, Matsuura Y, Miyamura T (1997). Sensitization to Fas-mediated apoptosis by hepatitis C virus core protein. Virology.

[43] Zhu N, Ware CF, Lai MM (2001). Hepatitis C virus core protein enhances FADD-mediated apoptosis and suppresses TRADD signaling of tumor necrosis factor receptor. Virology.

[44] Gao CF, Ren S, Zhang L, Nakajima T, Ichinose S, Hara T, Koike K, Tsuchida N (2001). Caspase-dependent cytosolic release of cytochrome c and membrane translocation of Bax in p53-induced apoptosis. Exp Cell Res.

[45] Li Y, Raffo AJ, Drew L, Mao Y, Tran A, Petrylak DP, Fine RL (2003). Fas-mediated apoptosis is dependent on wild-type p53 status in human cancer cells expressing a temperature-sensitive p53 mutant alanine-143. Cancer Res.

[46] Kao CF, Chen SY, Chen JY, Wu Lee YH (2004). Modulation of p53 transcription regulatory activity and post-translational modification by hepatitis C virus core protein. Oncogene.

[47] Jahan S, Khaliq S, Siddiqi MH, Ijaz B, Ahmad W, Ashfaq UA, Hassan S (2011). Anti-apoptotic effect of HCV core gene of genotype 3a in Huh-7 cell line. Virol J.

[48] Machida K, Cheng KT, Sung VM, Shimodaira S, Lindsay KL, Levine AM, Lai MY, Lai MM (2004). Hepatitis C virus induces a mutator phenotype: enhanced mutations of immunoglobulin and protooncogenes. Proc Natl Acad Sci U S A.

[49] Gannon HS, Woda BA, Jones SN (2012). ATM phosphorylation of Mdm2 Ser394 regulates the amplitude and duration of the DNA damage response in mice. Cancer Cell.

[50] Wei J, Ye C, Liu F, Wang W (2014). All-trans retinoic acid and arsenic trioxide induce apoptosis and modulate intracellular concentrations of calcium in hepatocellular carcinoma cells. J Chemother.

[51] Pratt MA, Niu M, White D (2003). Differential regulation of protein expression, growth and apoptosis by natural and synthetic retinoids. J Cell Biochem.

[52] Arora P, Kim EO, Jung JK, Jang KL (2008). Hepatitis C virus core protein downregulates E-cadherin expression via activation of DNA methyltransferase 1 and 3b. Cancer Lett.

[53] Lee CW, Sorensen TS, Shikama N, La Thangue NB (1998). Functional interplay between p53 and E2F through co-activator p300. Oncogene.

[54] Lansink M, van Bennekum AM, Blaner WS, Kooistra T (1977). Differences in metabolism and isomerization of all-trans-retinoic acid and 9-cis-retinoic acid between human endothelial cells and hepatocytes. Eur J Biochem.

[55] Kato T, Date T, Miyamoto M, Furusaka A, Tokushige K, Mizokami M, Wakita T (2003). Efficient replication of the genotype 2a hepatitis C virus subgenomic replicon. Gastroenterology.

[56] Zhong J, Gastaminza P, Cheng G, Kapadia S, Kato T, Burton DR, Wieland SF, Uprichard SL, Wakita T, Chisari FV (2005). Robust hepatitis C virus infection *in vitro*. Proc Natl Acad Sci U S A.

[57] Takeuchi T, Katsume A, Tanaka T, Abe A, Inoue K, Tsukiyama-Kohara K, Kawaguchi R, Tanaka S, Kohara M (1999). Real-time detection system for quantification of hepatitis C virus genome. Gastroenterology.

[58] Lim JS, Park SH, Jang KL (2011). All-trans retinoic acid induces cellular senescence by up-regulating levels of p16 and p21 via promoter hypomethylation. Biochem Biophys Res Commun.

[59] Herman JG, Graff JR, Myohanen S, Nelkin BD, Baylin SB (1996). Methylation-specific PCR: a novel PCR assay for methylation status of CpG islands. Proc Natl Acad Sci U S A.

